# Higher ultraviolet skin reflectance signals submissiveness in the anemonefish, *Amphiprion akindynos*

**DOI:** 10.1093/beheco/arac089

**Published:** 2022-11-01

**Authors:** Laurie J Mitchell, Fabio Cortesi, N Justin Marshall, Karen L Cheney

**Affiliations:** School of Biological Sciences, Faculty of Science, The University of Queensland, Brisbane, QLD 4072, Australia; Queensland Brain Institute, The University of Queensland, Brisbane, QLD 4072, Australia; Queensland Brain Institute, The University of Queensland, Brisbane, QLD 4072, Australia; Queensland Brain Institute, The University of Queensland, Brisbane, QLD 4072, Australia; School of Biological Sciences, Faculty of Science, The University of Queensland, Brisbane, QLD 4072, Australia

**Keywords:** Clownfish, color, contest, reef fish, social communication, vision

## Abstract

Ultraviolet (UV) vision is widespread among teleost fishes, of which many exhibit UV skin colors for communication. However, aside from its role in mate selection, few studies have examined the information UV signaling conveys in other socio-behavioral contexts. Anemonefishes (subfamily, Amphiprioninae) live in a fascinating dominance hierarchy, in which a large female and male dominate over non-breeding subordinates, and body size is the primary cue for dominance. The iconic orange and white bars of anemonefishes are highly UV-reflective, and their color vision is well tuned to perceive the chromatic contrast of skin, which we show here decreases in the amount of UV reflectance with increasing social rank. To test the function of their UV-skin signals, we compared the outcomes of staged contests over dominance between size-matched Barrier Reef anemonefish (*Amphiprion akindynos*) in aquarium chambers viewed under different UV-absorbing filters. Fish under UV-blocking filters were more likely to win contests, where fish under no-filter or neutral-density filter were more likely to submit. For contests between fish in no-filter and neutral density filter treatments, light treatment had no effect on contest outcome (win/lose). We also show that sub-adults were more aggressive toward smaller juveniles placed under a UV filter than a neutral density filter. Taken together, our results show that UV reflectance or UV contrast in anemonefish can modulate aggression and encode dominant and submissive cues, when changes in overall intensity are controlled for.

## Teaser

When signaling submission, it is important to wave the “white flag.” In the Barrier Reef anemonefish, ultraviolet color patterns serve to signal submissiveness. In staged contests over dominance between size-matched anemonefish, low ultraviolet skin reflectance strongly predicted fish winning a contest, while high UV skin reflectance predicted losing. We demonstrate that juvenile subordinates benefit by signaling their submissiveness with a naturally higher ultraviolet skin reflectance which evokes less aggression from larger, more-dominant fish and likely aids with social group integration.

## INTRODUCTION

Dominance hierarchies reduce the fitness costs of group living (Rowell 1974); however, for long-term stability, they require the coevolution of honest cues to clearly convey membership rank (Maynard-Smith and Harper 2003). Such “badges of status” can be relatively cheap to produce but their honesty is enforced by severe social costs imposed by receivers when they are an inaccurate indicator of an individual’s fighting ability or quality (Senar 1999; Searcy and Nowicki 2005; Ligon and McGraw 2016). Common cues used by animals for conveying dominance or submissiveness include changes in skin intensity (Höglund et al. 2000; Ligon 2014; Bachmann et al. 2017; Dickerson et al. 2020), aggression (Reeve and Nonacs 1997; Ang and Manica 2010), posturing (Allen 1975; Woo and Rieucau 2012; Reddon et al. 2019), and distinctive body color (Stuart-Fox and Moussalli 2008; Vedder et al. 2010; Martin et al. 2016; Names et al. 2019). By employing multiple components animals may also amplify cues to improve their saliency to intended receivers (Bokony et al. 2006; Harper 2006).

Abundant evidence shows that ultraviolet (UV) body colors and UV vision are widespread across non-mammalian vertebrates (Bennett et al. 1996; Jourdie et al. 2004; Siebeck 2004; Whiting et al. 2006; Roberts et al. 2009; Cronin and Bok 2016). While the exact energetic cost of UV signal production is unknown, it has been demonstrated to have pronounced social costs and to be an honest signal in some animals, for example, in birds (Keyser and Hill 2000; Griggio et al. 2009) and lizards (Whiting et al. 2006; Names et al. 2019). UV-signaling by teleost fishes may facilitate private communication over short distances, as UV light is rapidly scattered in aquatic habitats and most large predatory fishes are UV blind (Cummings et al. 2003; Siebeck 2004). Indeed, multiple studies have demonstrated the use of UV cues in assessing mate quality by small teleost fishes, for example, in guppys (*Poecilia reticulata*) (Garcia and de Perera 2002; Kodric-Brown and Johnson 2002; Smith et al. 2002), sword-tails (*Xiphophorus nigrensis*) (Cummings et al. 2003), and three-spined stickleback (*Gasterosteus aculeatus*) (Boulcott and Braithwaite 2005; Rick et al. 2006; Rick and Bakker 2008a). However, there remains scarce empirical evidence of the functions fish UV signaling has in other socio-behavioral contexts (but see Modarressie et al. 2005; Rick and Bakker 2008b; Siebeck et al. 2010), particularly for communication in social groups.

Anemonefishes (subfamily, Amphiprioninae) are a group of reef fishes that obligately inhabit one or more species of cnidarian anemones and are sequentially hermaphroditic (Allen 1975; Fricke and Fricke 1977). They live in strict dominance hierarchies maintained by differences in body size, where breeding is reserved to the largest (most-dominant) female and second largest (less-dominant) male, while any smaller fish (least-dominant) are sub-adult/juvenile subordinates (Fricke 1979; Buston 2003). Subordinate anemonefish forego reproduction in exchange for being granted shelter at anemones, and possible future breeding opportunities (Buston 2004; Buston and Cant 2006; Rueger et al. 2018). Dominant anemonefish enforce their rank by directing aggression toward the adjacent lower-rank, while the latter often respond by displaying submissive/appeasing postures (Allen 1975; Buston 2003; Iwata et al. 2008; Camp et al. 2016; Wong et al. 2017) and long term by stunting their growth rate (Buston and Cant 2006). Because of their small size and fragility, subordinates must maintain clear submissive signaling to reduce the risk of injury imposed by dominant fish and/or eviction from host anemones, usually resulting in death (Mariscal 1970; Allen 1975). All group members stand to benefit from the clear signaling of rank by also minimizing loss-of-opportunity costs and risk of lowered vigilance toward predators (Jakobsson et al. 1995). To what extent other possible cues (e.g., skin color or tone) have a role in anemonefish social communication remains unknown.

The distinctive appearance and conserved development of the orange and white bars of anemonefishes (Salis et al. 2018, 2019) has given rise to a variety of suggested functions, including for intra- and/or inter-species recognition (Fricke 1973; Buston 2003; Salis et al. 2018), camouflage, and aposematism for sea anemones (Merilaita and Kelley 2018). However, in a void of empirical testing, the function of their bard skin has remained elusive. Recently, it was shown that the white bars in the Barrier Reef anemonefish (*Amphiprion akindynos*) reflect strongly in the UV and that the fish have cone photoreceptors in the retina well suited to enhance the chromatic contrast between orange and white bars (Stieb et al. 2019). The high contrast of their UV colors combined with an apparent widespread nature of UV vision across anemonefishes (Mitchell et al. 2021), suggests a potentially conserved function in communication.

In this study, we behaviorally assessed the importance of UV signaling in social interactions between conspecific anemonefish (*A. akindynos*), specifically for conveying dominance and submissiveness in multiple life-stages, as preliminary data indicated differences in the UV reflectance between dominant and subordinate fish. Prior knowledge of their three cone spectral sensitivities (400, 498, and 520 λ_max_) (Stieb et al. 2019), and ritualized aggressive and submissive behaviors (Allen 1975) makes them ideal for testing the role of UV-signaling in social communication.

First, we conducted an experiment that compared the effect of altered UV-skin reflectance on the outcomes of staged contests over dominance between size-matched individuals. Ritualized agonistic and submissive behaviors were analyzed to determine the winners and losers of contest interactions between paired opponents viewed in opposing chambers with distinct light treatments induced by different UV-absorbing filters. Next, we tested the role UV signaling has in mediating conflict between hierarchical ranks, by comparing levels of aggression directed at smaller fish viewed in UV or UV-filtered light. To explore how the appearance of anemonefish skin compared across light treatments, we used computational models of *A. akindynos* color vision to estimate the chromatic contrast between their orange and white skin patches. We also modeled anemonefish skin contrast as viewed on the reef at a host-anemone to provide a reference for how any differences in skin reflectance likely appear in natural light conditions. These findings reveal a functional aspect of anemonefish skin patterns in dominance signaling and contribute to our broader understanding on the role coloration has in social communication.

## METHODS

### Animals and permits

Anemonefish collection and experiments were conducted during two separate field trips at the Lizard Island Research Station on the northern Great Barrier Reef, Australia between June–July 2019 and February–March 2021. We captured juvenile (standard length/SL range = 2.1–3.4 cm; *n* = 11), sub-adult (SL range = 3.6–5.6 cm; *n* = 15), male (SL range = 4.0–7.5 cm; *n* = 14), and female (SL range = 5.0–9.0 cm; *n* = 5) *A. akindynos* using hand-nets on snorkel or SCUBA from reef patches surrounding Lizard Island (GBRMPA permit no. G17/38160.1, Queensland Fisheries permit no. 180731 and 207976). Adults were identified as the largest (female) and second largest (male) fish on hosted anemones, while any smaller fish were classed as either sub-adult or juvenile based on visual characteristics (e.g., color, size), as per Allen (1975). The capture of males was prioritized over larger females (females often differ in size) to maximize the number of body-sized-matched pairings in our experiment, while using as few fish as possible. To minimize the impact of reduced anemonefish presence/defense on hosted sea anemones, we ensured that at least one dominant individual remained on each anemone at collection sites. Captured anemonefish were immediately transported back to the research station, where their standard length (SL) was measured using a caliper. They were then kept in individual flow-through aquaria (43.2 × 32.0 × 30.7 cm) that contained a PVC pipe for shelter until the start of a contest. Anemonefish were fed two times daily with a serving of pellets (Ocean Nutrition Formula One Marine Pellet). Post-experiment, all remaining fish were returned to their original anemones based on mapped locations and nearby tethered marker buoys. All experiments were conducted in accordance with The University of Queensland’s Animal Ethics Committee requirements (AEC approval no. 2016/AE000304 and 2017/AE000077).

### Experimental setup

**Figure 1:**
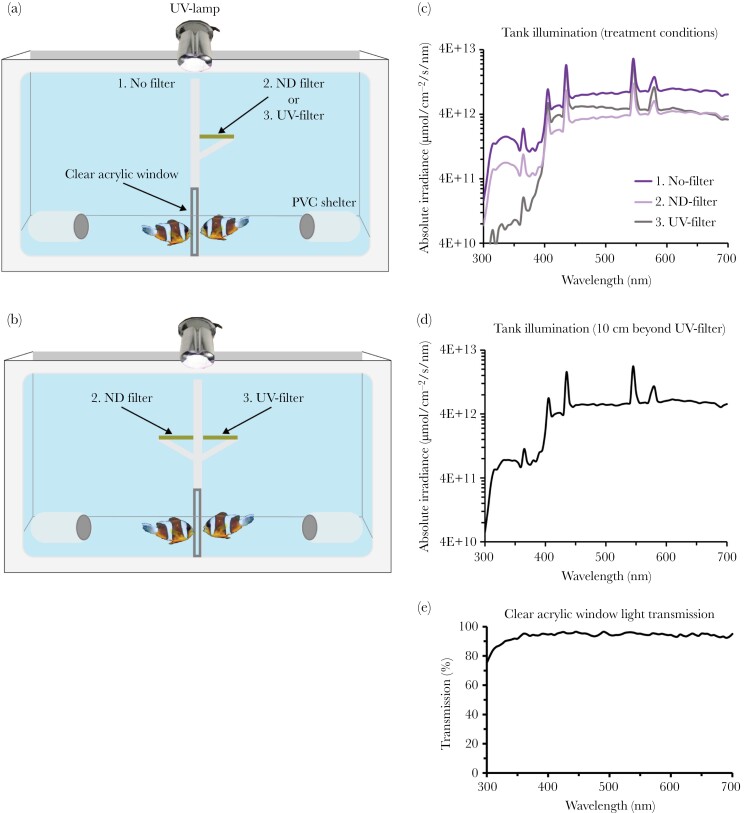
Illustrated side-views of experimental tanks in which anemonefish (*Amphiprion akindynos*) were shown under, including (A) no filter versus UV filter or ND filter, and (B) ND filter versus UV filter. (C) Measurements of side welling, absolute spectral irradiance (µmol/cm^2^/s/nm) under the three different light treatments, where an order of magnitude reduction in UV was induced by the UV filter. (D) 10 cm beyond the interaction/light-filtered area toward the center of the tank. (E) Measured spectral transmission of the clear acrylic window where anemonefish interacted. Note, that absolute irradiance is plotted in log-scale due to the peakiness in the visible range caused by the fluorescent light.

Behavioral experiments were conducted in three identical white tub aquaria fitted with a glass viewing panel on one side and a central opaque plastic divider that created two chambers (20.5 × 32 × 30 cm), which could be viewed from either side through a UV-transmissive acrylic window (15 cm^2^) (Figure 1A,B). Each chamber was illuminated by both indirect daylight and an overhead UV-lamp (Arcadia D3 ARC POD 11 Watt, 30% UVA compact lamp). The UV-lamp was necessary to compensate for the ambient illumination, where semi-transparent roofing cut daylight intensity most severely in the UV.

To investigate the role of UV-signaling in anemonefish interactions, we had three light treatments: 1) no-filter, 2) neutral density (ND) filter, and 3) UV-filter (Figure 1A,B). UV-filter and ND-filter light treatments were induced by constructing light-filter canopies (5.0 × 20.0 cm) that extended directly above the acrylic window and were comprised of UV filter (x4 LEE Filters 226 U.V.) and ND filter (x8 LEE Filters 130 Clear), respectively. We included a ND-filter treatment to control for the partial-absorption of light across the spectrum by the UV filters, allowing us to ascertain whether differences in the wavelength or intensity of reflected light were responsible for observed effects between light treatments, that is, whether the removal of UV alone or a general darkening of the body were having an effect. Light conditions were confirmed by measuring side-welling spectral irradiance directly under each light treatment using an Ocean Optics Jaz spectrometer fitted with cosine corrector, that was calibrated against a deuterium-halogen lamp (Mikropak DH2000-DUV, calibrated by Ocean Optics) (Figure 1C). Note, there was slight absorption of > 450 nm (in the blue–yellow region) under the UV-filter (Figure 1C), but this was found to have a negligible impact on skin chromatic contrast (±0.08–0.1 Δ*S* shift) and unlikely detectable by *A. akindynos* (full details on the visual modeling are given in Supplementary Figure 1).

Our canopy design ensured that changes in the available spectrum were mostly limited to areas adjacent to the window where anemonefish interacted. This minimized changes in the overall tank light environment (i.e., beyond the filters, see Figure 1D), an important consideration when making the distinction between behavioral responses toward altered anemonefish appearance from a possible reaction to changes in overall lighting. The clear acrylic window was fully transmissive across the visible spectrum of *A. akindynos*, including in the UV (Figure 1E). Minimal light spillage through the acrylic window contributed to trace levels of horizontal UV radiance detected in the UV-filter treatment (Figure 1C), that was more-readily detected when a (UV-reflective) PTFE sheet was held at a slight angle under either the no-filter or ND-filter treatment. Thus, we are confident of the reasonably strict light conditions in our experiment and that fish in the UV-filter treatment could still view the UV reflectance of opponents in the neighboring chamber.

### Experiment 1: effect of UV on staged contest outcome

To test the importance of UV-signaling in anemonefish interactions, fish were paired against size-matched opponents (*N* = 70 trials) within 1.0 cm SL (Δ_SL_ range = 0.0–0.85 cm; mean Δ_SL_ ± SD = 0.32 ± 0.24 cm). This criterion ensured that no opponents approached a size difference 1.26 times their body length, a critical size boundary between adjacent hierarchical ranks in family groups of a close relative, *A. percula* (Buston and Cant 2006). Fish were used in one to six contests, depending on the number of size-matched pairings (average number of contests per fish ± SD = 3.0 ± 1.5). No repeat pairings, nor pairs comprised of fish from the same anemone were made. Each pairing was pseudo-randomly assigned to one of three treatments (Figure 1A,B): 1) no filter versus UV filter (*n* trials = 21), 2) no filter versus ND (*n* trials = 15), and 3) ND versus UV filter (*n* trials = 34). At the beginning of each contest, fish from each pair were transferred by hand net into one section of the tank. They were given an adjustment period of 30 min with an opaque barrier placed in front of the acrylic window to prevent any pre-contest interactions. Immediately prior to contest commencement, the barrier was removed, and interactions were video recorded for 20 min using a Go-Pro (Hero 3) mounted on a tripod with a full view of the experimental tank. At 20 min, the contest was terminated, and fish were returned to their aquaria.

Analysis of the footage involved recording key agonistic and submissive behaviors (see Table 1) when anemonefish interacted with each other at the acrylic window. These behaviors are well documented in the literature (Allen 1975; Moyer and Bell 1976; Camp et al. 2016; Wong et al. 2017) and observed both on the reef and in captivity (pers. observ., 2019, 2020). Aggressive behaviors were classed as either attacks or intimidation, where attacks were considered behaviors that would have resulted in physical contact, including bites and charges, if a partition had not been present. Anemonefish-specific intimidatory behaviors included dorsal leaning: folding down of the dorsal fin and pivoting the body toward a recipient while either stationary or moving; head shaking: rapid quivering of the head to whole body while maintaining position in the water column; and head jerking/gaping: repetitive rapid jaw-movements (Table 1). Anemonefish also exhibit multiple submissive displays to avoid injury from more-dominant individuals including ventral leaning: folding down of the dorsal fin and pivoting the body away from the recipient; and head shaking, an identical behavior to the threat display but usually elicited by an attack (Allen 1975; Moyer and Bell 1976; Table 1). All these anemonefish behaviors were described in the highly detailed field observations of Allen (1975), and since corroborated by multiple studies focused on anemonefish social interactions (see Moyer and Bell 1976; Camp et al. 2016; Wong et al. 2017; Iwata et al. 2020). It should be noted that head jerking often coincides with vocalization, that is likely agonistic (Schneider 1964, reviewed by Allen 1975). On occasion, these were audible in video recordings; however, we were unable to reliably account for any sound cues in our study due to technical constraints. A previously unreported behavior was also observed in our experiment, which we termed “tail-slapping,” where fish rapidly wagged their caudal fin in front of recipients. This behavior usually resulted in an attack from the recipient, and therefore was interpreted as an agonistic display. Anecdotal evidence from captive *A. ocellaris* supports its agonistic role, where some fish direct this behavior at hands entering the aquarium during maintenance (pers. observ., 2020).

**Table 1:** Summary of aggressive and submissive anemonefish behaviors per staged contest (*N* = 70 contests, mean ± standard error)

Behavior		Description	Size-matched contest interactions (mean ± SE)	
			Winner	Loser
Aggressive	Bite (attack)	Biting directed at opponent against the window.	5.0 ± 0.5	4.0 ± 0.7
	Charge (attack)	Rapidly swimming toward opponent.	2.0 ± 0.2	2.0 ± 0.2
	Head jerking/gaping	Series of rapid jaw movements often coinciding with jaw clicking.	N = 10 (n = 7)	N = 5 (n = 5)
	Tail slapping	Facing away from and directing multiple consecutive slaps with tail at the opponent.	1.0 ± 0.5	2.0 ± 0.3
	Dorsal leaning	(Non-provoked) lateral or parallel mutual displays of folding down the dorsal fin and pivoting dorsal side toward opponent.	3.0 ± 0.4	3.0 ± 0.3
	Head shaking I	(Non-provoked) rapid quivering of the head.	3.0 ± 0.6	2.0 ± 0.3
Submissive	Ventral leaning	(Attack provoked) lateral or parallel displays of folding down the dorsal fin and pivoting the ventral side toward opponent.	-	80%
	Head shaking II	(Attack provoked) rapid quivering of head in direct response.	-	20%
	Hiding	Seeking shelter in PVC tube.	*N* = 9 (*n* = 6)	*N* = 19 (*n* = 11)

For behaviors with too few observations recorded (*N* ≤ 20), the raw count is given along with the number of contests in parentheses. Submissive postures, that is, one per contest, are expressed as the percentage out of all observations.

The outcome (winner and loser) of contests was decided by the first observation of submissive posturing displayed by either fish, subsequently deemed the loser. Because of the dual role that head shaking serves in agonism and submissiveness (Allen 1975; Moyer and Bell 1976; Camp et al. 2016; Iwata et al. 2020), and the occasional subtlety between dorsal leaning and ventral leaning, we avoided ambiguity by adhering to a strict criterion that postures were only submissive when immediately preceded by an attack (i.e., charge or bite) from the opponent. This closely resembled anemonefish interactions observed in nature, where larger fish reinforce their rank by charging at smaller fish who often immediately display either ventral leaning or head shaking instead of retaliating, causing the aggressor to terminate harassment (Allen 1975; Moyer and Bell 1976). Upon observing submission, the video time stamp (in seconds), and outcome (loser and winner fish IDs and corresponding treatments) was recorded and footage analysis was terminated. After the first submission, fish typically ceased interacting for the remaining duration of the contest, or interactions became infrequent, and the initial loser again displayed submission which supported our criterion. To further back our findings, the footage for 36 out of the 70 recorded contests were also spread between and observed by four volunteers in a blind analysis. Overall, we found seven discrepancies (out of 36) that did not match the contest outcomes from the initial analysis. To avoid potential observer bias, the seven contests that did not match initial outcomes were altered to reflect the blind analysis.

### Experiment 2: effect of UV on aggression directed at subordinates

To further investigate the potential role of UV signaling in anemonefish social interactions, we compared the effect of UV light and reduced UV light on the level of aggression directed by larger fish at smaller (less-dominant) fish. In this experiment, anemonefish pairings had a SL difference over 1.0 cm (Δ_SL_ range = 1.1 cm—3.5 cm; mean Δ_SL _± SD = 2.1 ± 0.72 cm), that exceeded the minimum threshold size difference between adjacent hierarchical ranks (Buston and Cant 2006). This perceived difference in rank was further emphasized by almost exclusively pairing sub-adults with juveniles. Prior to trial commencement, individual fish were randomly assigned (via coin toss) to either the UV filter or ND filter treatment and similar to the previous experiment were given a 30-min adjustment period with an opaque barrier placed in front of the acrylic window to prevent any prior interaction. Trials commenced after removing the opaque barrier and were video recorded for 20 min, after which video recording stopped and fish were returned to their aquaria.

Footage analysis involved counting the number of different agonistic behaviors (as per the staged-contest experiment, see Table 1) directed by the larger fish toward the smaller fish at the acrylic window. Behaviors exhibited by smaller fish were limited to typical submissive postures in response to attacks. Trial footage was analyzed for the full recorded 20 min (*N* = 20).

### Skin color measurement and analysis

**Figure 2:**
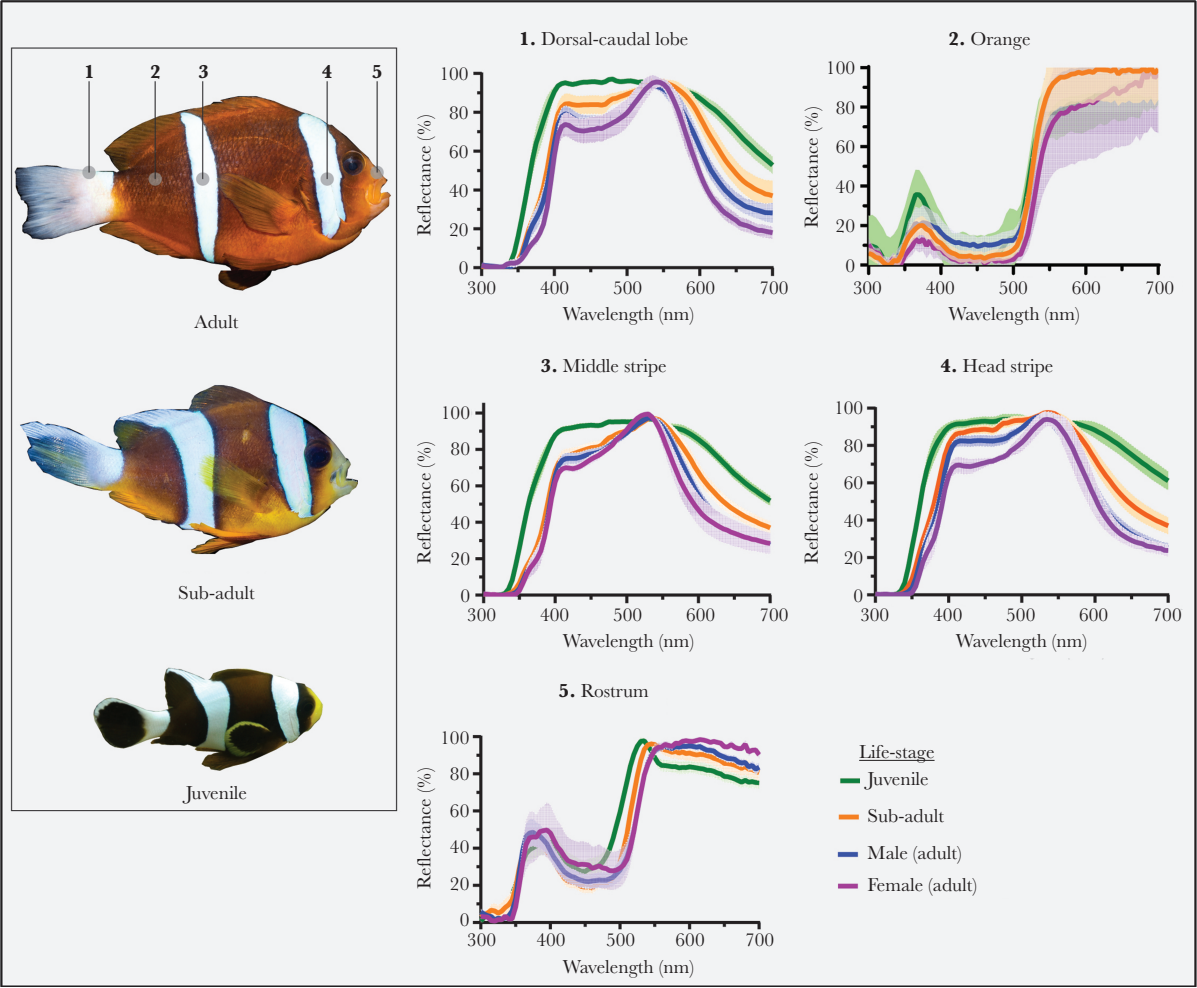
Skin spectral reflectance normalized to the highest intensity wavelength (%). Measurements were taken for five different *A. akindynos* skin patches plotted according to developmental stage including for adults (*n* males = 14, and *n* females = 4), sub-adults (*n* = 11), and juveniles (*n* = 10). Numbered labels indicate the order and approximate body location in which skin patches were measured, and correspond to a numbered subplot. Solid lines depict the average reflectance spectrum and shaded areas depict upper and lower s.e.m bounds. Note the consistency in the white color reflectance, including high ultraviolet, within and between patches (patches 1, 3, and 4) of juvenile fish. Image credit: photos of *A. akindynos* used with permission by Valerio Tettamanti.

After the behavioral experiments were completed, the spectral reflectance of multiple skin patches (Figure 2) was measured for anemonefish (*N* = 47) used in the experiment. Measurements were done across a 300–700 nm range using an Ocean Optics Jaz spectrometer (Ocean Optics, Largo, FL, USA) with pulsed Xenon light source (Jaz-PX module), and a bifurcated 200 µm fiber optic cable (Thorlabs, NJ, USA). Reflectance measurements were taken at a 45° angle against skin patches, relative to a Spectralon 99% diffuse reflectance standard (Lab-sphere, North Sutton, NH, USA). All measurements were taken out of water and in a dark room by two personnel (as per Marshall 2000a; Marshall et al. 2003). One person maintained the fiber in position above skin patches, while the second person took measurements and gradually exposed skin patches caudal-rostrally by rolling back a seawater-soaked towel that wrapped fish to protect their skin from desiccation and eyes from the bright light. We ensured consistency among measurements by targeting the same locations across anemonefish and compensated for changes in body contour/relief by adjusting the held distance of the fiber accordingly. Three measurements were taken per skin patch (within 0.5–1 cm^2^) and the average was used in subsequent analyses. To minimize stress from air exposure, all measurements were taken within 2 min per fish and no stress-related color changes were observed. Post-measurement, all fish promptly recovered in a bucket of seawater and were then returned to aquaria. A low number of measurements (39/225, ~17%) were discarded due to either being saturated or containing artifacts.

To determine whether the color contrast of anemonefish in our laboratory experiments would appear similar when viewed by conspecifics in their natural habitat, we used the Receptor Noise Limited (RNL) model (Vorobyev and Osorio 1998) to calculate the chromatic contrast of the rostrum and multiple white skin patches against adjacent orange skin in anemonefish viewed in our experimental light treatments and in reef illumination at 5 m depth (for further details, see Supplementary Materials). We also determined the color contrast between fish color patches and a host sea anemone. The RNL model combines visual system parameters with the light environment to measure color distance in units of Δ*S*, where the minimum perceivable difference between two objects under well-lit conditions is assumed to be a value of Δ*S* = 1 (Vorobyev and Osorio 1998).

To model anemonefish skin colors, receptor quantum catches “*q*_*i*_,” were first calculated for each photoreceptor type “*i*” (based on equation 1 from Vorobyev and Osorio 1998) by,


$$\begin{array}{l} q_{i}=k_{i}\underset{700}{\overset{300}{\int }}S_{i}\left(\lambda \right)R\left(\lambda \right)I(\lambda )\;d\lambda , \end{array}$$ (1)

where *S*_i_(*λ*) was the spectral sensitivity of a given photoreceptor type “*i*” (*i* = SWS, MWS, LWS) already multiplied by lens transmittance, and “*λ*” denoted wavelength (nm). We used the cone spectral sensitivities of *A. akindynos* (400, 498, and 520 λ_max_) as reported by Stieb et al. (2019). “*R*(λ)” was the normalized reflectance spectrum of anemonefish skin, “*I*(λ)” was the illumination spectrum as absolute sidewelling spectral irradiance (µmol/cm^2^/s/nm) in experimental light treatments (no filter, UV filter, ND filter), and “*k*_*i*_” was the von Kries scaling factor for photoreceptor adaptation to the background or color constancy (Chittka et al. 2014), as calculated by:


$$\begin{array}{l} k_{i}{=}^{1}{/}_{\underset{700}{\overset{300}{\int }}\;S_{i}\left(\lambda \right)\;R\left(\lambda \right)\;I^{b}\left(\lambda \right)\;d\lambda } \end{array}$$ (2)

where *I*^*b*^(λ) was the background spectrum, as the side welling irradiance measured inside the aquarium away from the light manipulating canopies. Integration was performed across the visible spectrum (i.e., 300–700 nm for *A. akindynos*).

Next, the contrast (Δ*q*_*i*_) for each receptor channel was calculated as the log of the quantum catch ratio by,


$$\begin{array}{l} \Delta q_{i}=ln\frac{q_{i_{1}}}{q_{i_{2}}} \end{array}$$ (3)

where “*qi*_*1*_” was the relative quantum catch of receptor type “*i*” for white skin patches (Figure 2), and “*qi*_*2*_” was the relative quantum catch of the adjacent orange skin.

In the absence of direct noise measurements for *A. akindynos* cones, we estimated cone noise levels (*e*_*i*_) by,


$$\begin{array}{l} e_{i}=\sqrt{\frac{\sigma }{\eta_{i}}}\; \end{array}$$ (4)

where “σ” (the numerator of the Weber fraction in Olsson et al. 2015, or “*v*_*i*_” in Vorobyev and Osorio 1998) is the standard deviation of noise within a photoreceptor, and “η” is the ratio or relative abundance of a given photoreceptor type in the retina. We set a σ-value = 0.1 and η of SWS, MWS, LWS = 1: 2: 2, that were identical to previously estimated values for *A. akindynos* cones (Stieb et al. 2019).

We then combined calculated values of Δ*q*_*i*_ and *e*_*i*_ from across all three cone types in *A. akindynos* to calculate Δ*S* for plotting in a trichromatic visual space using:


$$\begin{array}{l} \Delta S=\sqrt{\left(\frac{e_{1}^{2}{\left(\Delta_{q3}-\;\Delta_{q2}\right)}^{2}+\;e_{2}^{2}{\left(\Delta_{q3}-\;\Delta_{q1}\right)}^{2}+\;e_{3}^{2}{\left(\Delta_{q1}-\;\Delta_{q2}\right)}^{2}}{{\left(e_{1}\;e_{2}\right)}^{2}+\;{\left(e_{1}\;e_{3}\right)}^{2}+\;{\left(e_{2}\;e_{3}\right)}^{2}}\right)} \end{array}$$ (5)

### Statistical analyses

Unless otherwise mentioned all statistical analyses and color vision modeling were conducted using the statistical program R v. 4.0.2 (R Core Team 2021).

To assess the effect of light treatment on staged-contest outcome, we ran binomial family generalized linear mixed effects models (GLMM) with log-link function for each of our three combinations of experimental light treatments (modeling scenarios: 1 = no-filter vs. UV-filter; 2 = UV-filter vs. ND-filter, and 3 = no filter vs. ND filter) using the “glmer” function in the package “lme4” v.1.1-27 (Bates et al. 2015). GLMMs included contest outcome (win = 1, loss = 0) as a binomial response, while light treatment (no-filter, UV-filter, and ND-filter), difference in body size (“delta_SL”), counts of prior wins/losses, developmental state (juvenile, sub-adult, male, female), and total count of aggressive behaviors (bites, charges, dorsal leaning, and body shakes) were held as fixed predictor variables. Developmental state was later dropped as a fixed effect variable due to its absence in all retained informative models. “Fish ID” was included as a random effect to account for individual variation. All global models reported to have successfully converged using the default “Nelder_Mead” optimizer. Originally, we also tried to include “Contest ID” as a random effect to account for the repeated use of anemonefish; however, the data could not support a model of such complexity and produced overfitted (i.e., singular) models. Thus, we removed “Contest ID” as a random slope to obtain a more parsimonious model (as recommended by Barr et al. 2013).

An alternative test of whether the repeated use of anemonefish strongly influenced future contest outcome, involved a small number (*n* = 6) of rematches between fish under swapped light treatments to their original contest. No repeated outcomes (wins/losses) were observed (for a summary of the rematches see Supplementary Table 1), providing evidence that the outcome of future contests was not influenced by the outcome of previous contests.

Based on the global GLMM for each modeling scenario, we used the “dredge” function from the package “MuMIn” (Barton 2020) to select the best-fitted models out of all possible combinations of variables according to highest corrected Akaike information criterion (AICc), and then performed model averaging across those within < 4 AICc differences (ΔAICc) of the best. Typically, models within 2 ΔAICc are considered as good a fit as the best model but depending on confidence level, the models slightly above this range can be considered (Symonds and Moussalli 2011; Burnham and Anderson 2016; Dickerson et al. 2020). In our case, GLMMs across all three scenarios had confidence of between 58.0% and 74.0% (cumulative AICc weights = 0.58–0.74), and we improved our chances of retaining the best model by considering models with ΔAICc < 4 (cumulative AICc weight shifts = 0.76–0.90). Any nested models which differed by one parameter from the best model and did not improve the log-likelihood were dropped to avoid elevating the weight of non-informative parameters from model averaging (Arnold 2010). Model-averaged coefficients and 95% confidence intervals were then calculated across retained models (function “model.avg” from package “MuMIn”; Barton 2020). A measure of the relative importance of variables (RIV) was calculated by summing normalized AICc weights across retained models which contained a variable of interest, as per Symonds and Moussalli 2011 and Dickerson et al. 2020.

To compare the effect of UV light on larger fish aggression, we ran a Poisson family GLMM (function “glmer” from package “lme4”; Bates et al. 2015) with the grand total count of aggressive behaviors (bites, charges, dorsal leaning) as the response variable, while smaller fish light treatment (UV filter, ND filter), fish body size difference (ΔSL), small fish life stage (state 1), and large fish life stage (state 2) were entered as fixed predictor variables. Fish IDs for both the UV-filtered treatment (fish ID 1) and ND treatment (fish ID 2), and trial number (trial ID) were included as random effects to account for the repeated use of fish. As per the analysis of Experiment 1, we selected the best-fitted models according to highest AIC, and then performed model averaging using top retained models (< 4 ΔAICc of the best) that lacked non-informative parameters (cumulative AICc weight = 0.96). Pairwise comparisons using the Kruskal–Wallis Test (“kruskal.test” in “stats” package; R Core Team 2021) then assessed for statistically significant differences between counts of specific agonistic behaviors directed at smaller fish under ND-filter and UV-filter treatments.

Quantum catches and color distances (Δ*S*) of anemonefish skin patch measurements were calculated using the log-RNL model within the trichromatic space of *A. akindynos* using the functions “vismodel” and “coldist” in the package “PAVO” (Maia et al. 2019).

One-way ANOVA followed by Tukey multiple comparisons (Graphpad Prism v.8.3.0) assessed for statistically significant (*P *< 0.05) differences among individual RNL modeled Δ*S* values of skin patches across different anemonefish life-stages.

## RESULTS

### Experiment 1: effect of UV on staged contest outcome

Anemonefish readily interacted during trials and often immediately displayed agonistic behaviors at the acrylic window. Contest duration ranged from 5 to 860 s (mean ± SE = 113 ± 20 s) until either fish displayed submission. Only in one contest did neither fish exhibit submission, for which the outcome was indeterminable (i.e., a tie) and the contest was excluded from analysis. Light treatment was the strongest predictor of contest outcome in all models for both no filter versus UV filter (scenario 1 in Table 2; *n* = 1 retained model in top subset; binomial GLMM: *z* = 2.25, SE = 1.04, *P* = 0.023), and the UV filter vs. ND filter (scenario 2 in Table 2; *n* = 5 models retained in top subset; binomial GLMM: *z* = 3.14, SE = 0.56, *P* = 0.002) contests (both RIVs = 1.00; Table 2), where anemonefish in the UV-filter treatment won 76.0% (*N* = 21) and 71.0% (*N* = 33) contests, respectively. Whereas light treatment was a weak predictor in models for the no-filter versus ND-filter contests (scenario 3 in Table 2; RIV = 0.14, in one out of five retained models), where there was an almost even split in the number of won contests between fish in either light treatment (no-filter treatment wins = 47.0%, *N* = 15). Thus, it seems a reduction in UV had a strong effect on the outcome of contests and improved the probability of fish winning.

**Table 2:** Model-averaged effect sizes of parameters that predicted the outcome of staged anemonefish contests with different light treatment combinations (model term)

Model term	Model-averaged estimate coefficient	Model-averaged 95% CI	RIV	*P*-value
1. No-filter—UV-filter Parameters				
Intercept	1.17	**0.23, 16.05**	-	0.058
Light treatment	−2.34	−**4.37,** −**0.30**	**1.00**	**0.023**
ΔSL		**-**	-	-
Prior wins	-	-	-	-
Prior losses	-	-	-	-
Aggression	-	-	-	-
2. UV-filter—ND-filter Parameters				
Intercept	−0.89	−**1.67,** −**0.12**	-	**0.024**
Light treatment	1.78	**0.67, 2.90**	**1.00**	**0.002**
ΔSL	−0.51	−0.90, 0.41	0.44	0.099
Prior wins	0.05	−0.16, 0.17	0.09	0.870
Prior losses	−0.16	−0.23, 0.19	0.1	0.574
Aggression	0.01	−0.18, 0.21	0.1	0.904
3. No-filter—ND-filter Parameters				
Intercept	0.05	−1.07, 1.18	-	0.926
Light treatment	−0.31	−0.73, 0.65	0.14	0.732
ΔSL	0.26	−0.43, 0.54	0.21	0.586
Prior wins	-	-	-	-
Prior losses	−0.55	−1.03, 0.74	0.26	0.460
Aggression	-	-	-	-

Effect sizes are given for fixed variables from top GLMMs (ΔAIC_c _< 4) for contests in scenario 1 (*n* = 21), scenario 2 (*n* = 34), and scenario 3 (*n* = 15) with 95% confidence intervals (CI). A relative importance value (RIV) of 1.00 indicates the parameter occurred in all top models.

-, parameter missing from models; ΔSL, difference in fish standard length (mm). Statistical significance of *P* < 0.05 is indicated in bold.

Other variables including difference in body size (ΔSL), number of prior wins and losses, and aggression (i.e., sum of bites, charges, agonistic postures) were poor predictors of contest outcome (RIVs = 0.00–0.61; Table 2). ΔSL weakly predicted contest outcome in modeling scenario 2 (RIV = 0.61; retained in the best model) but this interaction was not statistically significant (binomial GLMM: *z* = 0.89, SE = 0.34, *P *= 0.373). To be certain of no treatment dependent influence on ΔSL, we also compared the distribution of ΔSL between light treatments in all modeling scenarios and found no signs of a bias in the allocation of larger/smaller fish pairings (Wilcoxon signed rank test, *P *> 0.05; Supplementary Figure 2).

In repeated contests (*n* = 6) which were excluded from the main analysis (Supplementary Table 1), there was no evidence that experience (previously winning/losing) or that the life stage/sex of fish determined victory. Males (Fish 9 and 2) in the UV-filter chamber initially won against similar sized females under no filter (Fish 6 and 7), and then lost against the same female in the repeat contest when the light treatment was swapped.

Note that the state/sex of fish in contests was consistently absent from the retained informative models, and therefore, it was removed as a variable from our analyses. See Supplementary Tables 2–7, for global model outputs and individual averaged model effects.

### Experiment 2: effect of UV on aggression directed at subordinates

**Figure 3:**
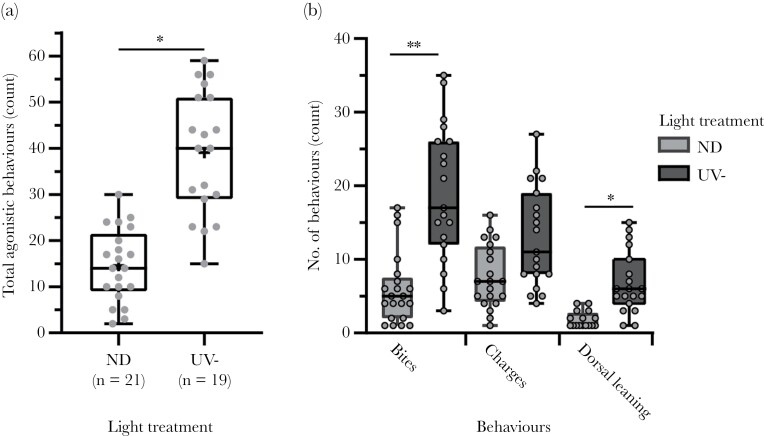
(A) Total observed (counts) of agonistic behaviors and (B) counts of individual agonistic behaviors directed toward smaller fish in neutral density filter (“ND”) and UV-filter (“UV-”) light treatments. Gray points denote individual counts per trial also given as the sample size. Box plots show the median, 25th and 75th percentiles, and range indicated by the whiskers. “*” and “**” denotes statistical significance of *P* < 0.05, and *P *< 0.01, respectively.

To further explore the role of UV-signaling in anemonefish interactions, we compared the number of aggressive responses directed at smaller fish shown under the UV-filter (*n* = 19) and ND-filter (*n* = 21) treatments. Larger fish during trials were consistently observed to be the instigator of interactions at the acrylic window and the sole aggressor, while smaller fish responded consistently with submissive postures. The light treatment of the smaller fish was the strongest predictor of the total count of aggressive behaviors from larger fish in nearly all retained models (RIV = 0.92, *n* = 4 retained models out of *N* = 5; Table 3), where smaller fish in the UV-filter treatment received on average 2.6 times the number of agonistic behaviors (mean ± SE = 39 ± 3.0 behaviors; Figure 3A) than in the ND-filter treatment (15 ± 2.0 behaviors, Poisson GLMM: *z *= 2.14, SE = 0.36, *P* = 0.033; Figure 3A). We also observed that agonistic behavior directed at smaller fish in the UV-filter treatment tended to persist immediately after submissive posturing during interactions (13/19 trials, 68%), while usually ceased after submission by smaller fish in the ND-filter treatment (17/21 trials, 81%). All remaining variables of fish SL difference (ΔSL) and state poorly predicted aggression in trials (Table 3). See Supplementary Tables 8 and 9 for the global model output and individual averaged model effects.

**Table 3:** Model-averaged effect sizes of parameters that predicted the total count of aggressive behaviors directed at smaller fish in trials (*N* = 40)

Model term	Model-averaged estimate coefficient	Model-averaged 95% CI	RIV	*P*-value
Total aggression Parameters				
Intercept	2.71	**2.24, 3.19**	-	**<0.0001**
Small fish treatment	0.79	**0.07, 1.51**	**0.92**	**0.033**
Δ_SL_	−0.08	−0.29, 0.13	0.55	0.445
Fish 1 state	0.02	−0.20, 0.25	0.10	0.833
Fish 2 state	−0.02	−0.24, 0.20	0.09	0.857

Effect sizes are given for fixed variables from top GLMMs (ΔAIC_c _< 4) for behavioral assays quantifying aggression by larger fish toward smaller fish in ND-filter and UV-filter treatments with 95% confidence intervals (CI). A relative importance variable (RIV) of 1.00 indicates the parameter occurred in all top models.

ΔSL, difference in fish standard length (mm). Statistical significance of *P* < 0.05 is indicated in bold.

A post hoc analysis of the different aggressive behaviors recorded in trials showed the majority were bites, charges, and dorsal leanings. Smaller fish in the UV-filter treatment received significantly more bites (Kruskal–Wallis test: *z *= 4.48, *P* = 0.0001; Figure 3B) and dorsal leanings (Kruskal–Wallis test: *z *= 3.21, *P* = 0.02; Figure 3B) than in the ND-filter treatment.

Tail slapping was a novel behavior observed in our experiment, that although rarely observed (*N* = 20 observations) was near exclusively directed at smaller fish in the UV-filter treatment in five out of six trials. Strangely, tail slapping was a behavior not previously characterized in the literature. We considered the possibility this was elicited by the artificial conditions in our experiment, and therefore, took a conservative approach by excluding tail slapping in our total counts of aggression. Gaping and head shaking were also rarely observed (*N* < 5 observations) and limited to trials where smaller fish were in the UV-filter treatment; however, because of their infrequent occurrence both were dropped from our analysis.

### Skin color analysis

**Figure 4:**
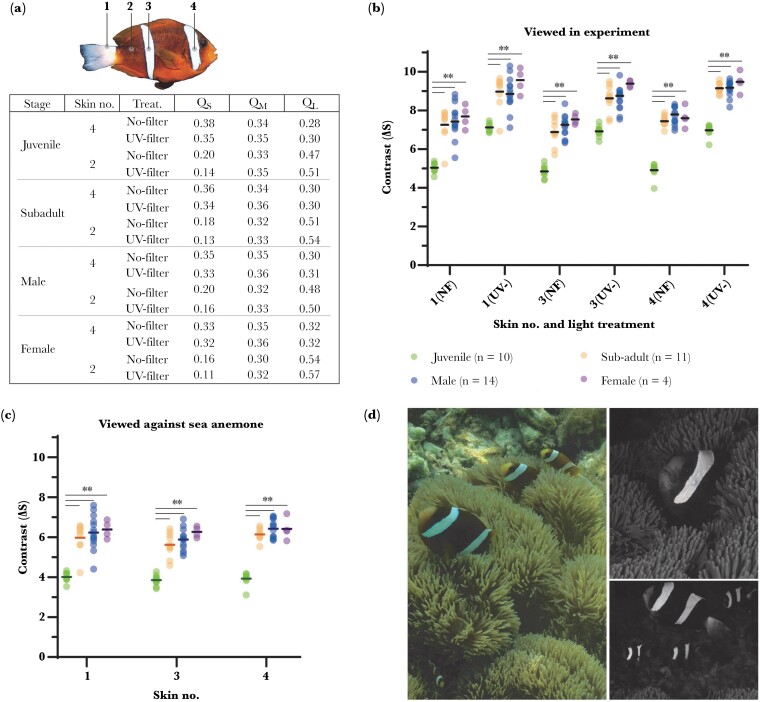
(A) Relative average cone quantum catches (Qi) of anemonefish head bar (skin no. 4) and orange skin (skin no. 2) viewed in the no-filter and UV-filter treatments. For presentation purposes, Q_i_ are only given for the head bar, as there was minimal variation (difference in Q_i_ ≤ 0.02) among white skin areas. For a full summary of skin Q_i_ values, see Supplementary Table 8. (B) Calculated chromatic contrast (Δ*S*) for different anemonefish white skin relative to orange skin when viewed in the no-filter (“NF”) and UV-filter (“UV-”) treatments, or (C) in natural reef illumination against host sea anemone tentacles. Note, the analysis of rostrum spectra was not included due to the high variability of measurements which made it difficult to accurately compare contrast. “**” denotes a *P*-value < 0.001. (D) Underwater photographs taken of *A. akindynos* at host sea anemones in RGB/color (left panel) and UV-only (monochrome, right panels). For details of the camera setup, see Supplementary Materials.

To examine for changes in the appearance of *A. akindynos* skin induced by our experiment, we quantitatively modeled the chromatic contrast of white skin (bars and caudal peduncle) against adjacent orange skin in each light treatment. Overall, SWS cone stimulation was highest for the skin patches of juveniles (Figure 4A), particularly for their more achromatic white skin patches (i.e., bars and caudal peduncle). Juvenile white skin had shorter UV-reflectance (mean 50% wavelength reflectance ± sd ≈ 361 ± 10.0 nm; see Figure 2) than sub-adults (387 ± 11.0 nm), males (389 ± 10.6 nm), and females (394 ± 5.4 nm). Together, the less-saturated orange of juveniles meant contrast between their white skin and adjacent orange skin (5.0 ± 0.3 Δ*S*; Figure 4B) was significantly lower, i.e., the bars appear more similar (ANOVA: all *P *< 0.001; Figure 4B) than equivalent areas in females (7.6 ± 0.4 Δ*S*), males (7.5 ± 0.7 Δ*S*), and sub-adults (7.2 ± 0.6 Δ*S*). An MWS/LWS shift in anemonefish skin color was induced by the UV filter which reduced SWS cone stimulation. This shift in lighting raised the contrast of white skin against orange skin by ~1.5 Δ*S* to ~2.0 Δ*S* across all life stages (Figure 4B), an effect caused by the reduced spectral reflectance overlap between white and orange skin in the UV-region (~375 nm). In juveniles, white–orange skin contrast (7.0 ± 0.3 Δ*S*; Figure 4B) was raised to near-match that of other life stages under no filter. No differences in skin patch Δ*S* were detected between the full light and dim treatments (Supplementary Table 10), a confirmation that our dim treatment affectively controlled for intensity without altering the chromatic component of skin color.

Overall, relative differences in modeled skin color appearance between hierarchical rank were consistent between our experiment (no filter) and natural reef lighting (Figure 4C). On coral reefs, the UV-contrast of anemonefish white bars confers a bold appearance amidst orange skin when viewed against a background of sea anemone tentacles (Figure 4C,D). When viewed in natural reef light, the white bars of juveniles also stimulate SWS cones stronger than in other life stages and consistently have lower contrast against orange (juveniles = 4.0 ± 0.3 Δ*S*, subadults/adults ≈ 6.0 Δ*S*; ANOVA: all *P *< 0.001, Figure 4C).

## DISCUSSION

UV signaling in many teleost fishes appears to be used for short-range social communication. However, few studies have investigated its role in social interactions beyond mate selection in fishes (but for territoriality see, Rick and Bakker 2008b; Siebeck et al. 2010; and for shoaling, see Modarressie et al. 2005). We have shown the amount of UV reflectance in *A. akindynos* skin declines in post-juvenile stages of development, and the strength of UV-skin reflectance strongly influenced the outcome of staged-contests for social dominance when difference in body size was not a factor. In experiment 1, when UV cues were diminished by placing fish under a UV-filter contests were more often won against fish under no filter or ND filter. However, there was no meaningful difference in the proportion of wins for contests between the no-filter and ND-filter treatment, with the latter reducing luminance of skin colors across the spectrum. Therefore, it was the chromatic component of the UV color rather than differences in luminance that fish responded. Separate from the main experiment, a subset of repeated contests further showed the importance of anemonefish UV reflectance in deciding victory, regardless of experience and other factors (e.g., life stage/sex, minor discrepancy in body size). In experiment 2, we found that anemonefish were more aggressive toward smaller (i.e., less-dominant) fish shown under the UV filter than ND filter, thus, punishing smaller fish that falsely advertised their status. Considering these findings, we propose that UV-skin patterns may serve in recognizing the distinct UV/white colors of juveniles that signal submissiveness.

### Effect of UV on staged contest outcome

Many animals possess conspicuous color traits to signal fighting ability in contests benefiting all participants by enabling the rapid assessment of expected contest outcome and avoidance of overly costly interactions (Rohwer 1975). It is well established that anemonefish dominance hierarchies are maintained by strict differences in body size from the most-dominant (largest) adults to least-dominant (smallest) subordinates (Buston 2003; Buston and Cant 2006). Results from our study suggest that higher UV reflectance in *A. akindynos* skin may signal submissiveness, as inferred by how its non-altered state strongly predicted a loss in staged contests. By extension, the inverse likely applies that lower skin UV reflectance was interpreted as a sign of dominance. Additional support for this idea comes from fish in opposing light chambers being similarly aggressive, indicating that victory was decided by fish behaving more submissive in response to opponents which reflected less UV, rather than being more aggressive toward an opponent who reflected more UV. It is unclear whether anemonefish recognized UV-dark fish as more dominant through experience at host anemones or were aware of their own UV reflectance. Although this cannot be explained alone by chromatic contrast between orange and white skin, due to only minor discrepancies (<1 ΔS) among post-juveniles (sub-adults and adults) and could simply be a reaction to difference in color signal. However, only three subadults and adults were collected from the same anemone, and therefore, we cannot rule out differences in skin reflectance that occur at the group level. A comparison of post-juvenile fish skin appearance within social groups could help further resolve whether UV-skin patterns can be used in recognizing the hierarchical rank of individuals.

Juvenile white skin both when viewed in our experiment and at host anemones was indicated to have noticeably lower contrast against adjacent orange skin (by ~2ΔS) that likely gave their white bars a distinct appearance from those of older life stages. This difference in juvenile appearance was determined to be caused by stronger UV reflectance in their white skin and to a lesser extent orange skin that combined reduced white–orange contrast. These findings expand upon those of Stieb et al. (2019) that similarly reported UV contrast in *A. akindynos* skin but did not detect any differences with juveniles, a detail likely missed by having only one sample per life stage. Our UV-filter treatment accentuated the contrast of the white against orange skin in all life stages, for which most notably in juveniles was shifted to roughly equate that of sub-adults/adults viewed under no filter. Interestingly, the behavioral effect induced by light treatment persisted in contests among sub-adults and adults, despite no differences in their base skin reflectance, a possible indicator of its generalizability in our experiments.

### Higher UV reflectance as a submissive signal in juveniles

The idea of a submissive UV signal was supported by our second experiment that showed larger anemonefish (mostly sub-adults) aggression toward smaller anemonefish (mostly juveniles) was heavily influenced by whether UV contrast was altered. This large difference in agonistic response than compared to the first experiment indicates that the size difference between fish in the second experiment was sufficient to perceive a clear difference in social rank. Juvenile anemonefish are the least-dominant member(s) of family groups and must overtly display their submissiveness to avoid incurred costs from harassment, injury and/or eviction from anemones by larger fish (Allen 1975). Due to the urgency of seeking shelter and patchiness of sea anemones as a habitat, recently settled juveniles often risk aggression by joining pre-established groups (Fautin 1992). This UV-submissive signal could aid in mitigating unnecessary conflict and the integration of juveniles into family groups. Indeed, other territorial reef fishes have unique skin color and patterns during their juvenile stage (i.e., paedomorphism), which aid in minimizing intraspecific conflict with adults, e.g., emperor angelfish (*Pomacanthus imperato*r) (Fricke 1980), threespot damselfish (*Stegastes planifrons*) (Thresher 1977), and Ambon damselfish (*Pomacentrus amboinensis*) (Gagliano 2008). Thus, it is feasible that juvenile anemonefishes and possibly other damselfishes use UV colors as a submissive signal to adults.

Although we detected consistent differences in the skin reflectance and appearance of anemonefish in our experiment, we were unable to empirically assign a function to any specific skin patch owing to the whole-body changes in appearance induced by our light treatments. A follow-up experiment that selectively manipulates different UV-reflective areas (e.g., by topical masking, as per Garcia and de Perera 2002) is necessary to assign the submissive signal to one or more skin pattern element(s) (i.e., head bar, middle bar, caudal peduncle).

Interestingly, our results show the direct opposite relationship between the amount of UV reflectance and perceived dominance status than reported in other animals, such as in flat lizards (*Platysaurus broadleyi*, Stapley and Whiting 2006; Whiting et al. 2006), lacertid lizards (*Podarcis muralis*, Names et al. 2019), blue tits (*Cyanistes caeruleus*, Roberts et al. 2009), and damselflies (*Megaloprepus caerulatus*, Xu and Fincke 2015). To our knowledge, this is the first evidence of a submissive UV signal in any animal. One possible explanation for this difference could be that UV reflectance is cheap to produce by anemonefish, hence its utility as a submissive signal by juveniles. The white skin of early-juvenile false clownfish, *A. ocellaris*, is comprised of iridophores, and to a lesser extent melanophores (Salis et al. 2018, 2019). As opposed to the pigmentary melanophores, iridophores provide structural coloration known to confer iridescence and UV reflectance (Cott 1940). Presumably, a small number of iridophores also underly orange skin in *A. akindynos* that reflect relatively low amounts of UV. Pharmacological treatments that knockdown iridophore development (e.g., TAE 684 treatment, as per Salis et al. 2018) could help resolve the cellular basis of UV reflectance in anemonefish skin. While the exact energetic cost of iridophore production and UV coloration in general is unknown, that of melanization can be considerable (Andersson 1994; Talloen et al. 2004). An increased abundance of melanophores (melanisation) with age may explain the lower UV reflectance of post-juvenile *A. akindynos* skin, which may serve as an honest signal of dominance. Alternatively, it is plausible that higher social costs from aggression by adjacent higher ranks have a greater role in maintaining signal honesty than energetic costs or predation risk.

### UV reflectance as part of multicomponent signaling

One outstanding question is why anemonefish would use multiple visual cues (i.e., posturing, body size, and UV colors) for signaling submission? Strong learning-effects likely underlie built-associations between skin coloration and submissiveness, as is the case for threespot damselfish which exhibit a bright yellow skin coloration as juveniles that elicits harassment in the short-term from darker adults, but the severity and frequency of which ease in the long-term permitting juveniles to establish new territories (Thresher 1977). Many animals use multiple signals to convey a single message, that by various learning mechanisms (e.g., emergent messaging, back-up cues, attention grabbing) aim to ensure the reliability and saliency of communication (reviewed by Candolin 2003; Bro-Jørgensen 2010). Based on the finding that smaller fish received increased attacks and threat displays when UV-signals were tampered with, it is apparent that differences in *A. akindynos* coloration acted in-tandem with body size for signaling submissiveness. We speculate that multiple visual components in juveniles serve to amplify submissive signaling by enhancing its learnability to sub-adults/adults. Such redundancy in signaling can enhance the learning efficiency of receivers in discrimination and learning-based tasks (Rowe 1999). Similar learning mechanisms have been shown in damselfly dominance signaling, where UV wing coloration amplifies perceived body size (Xu and Fincke 2015); however, this does not appear to always be the case (e.g., in lacertid lizards, Names et al. 2019).

Note, our study did not account for potential color change induced by body movement or dynamic pigment changes. Because of technical constraints our footage analyses of anemonefish interactions were limited to the visible range recordable by off-the-shelf cameras. Submissive ventral leaning and head-shaking postures in anemonefish may also serve to accentuate the white bars by optimizing the light angle of incidence relative to the receiver and introducing an aspect of motion, respectively. The latter has been similarly suggested for the head-bobbing behavior and dewlap coloration of many lizards (Ord et al. 2015). The finding that aggression toward smaller fish often persisted under the UV filter even after submissive posturing, while ceased after submission by smaller fish under the ND filter suggests an interplay of motion and color in signaling submission. Further investigation of anemonefish interactions using a multi-channel, UV/visible-imaging camera setup could reveal the use of UV signaling in dynamic submissive displays.

### Is UV communication widespread in anemonefishes?

Whether these results found in *A. akindynos* reflect a more general function of anemonefish skin appearance is unknown, but interestingly multiple anemonefish species exhibit supplementary white bars as juveniles that are subsequently lost toward the beginning of the sub-adult phase (e.g., *A. melanopus, A. frenatus, A. mcculochi, A. rubrocinctus*; Allen 1975), possibly hinting at its conserved importance in juvenile signaling. Variation in the number of bars has also been suggested to be a means of reducing conflict and supporting cohabitation both within social groups and among species (Salis et al. 2018). Other unrelated suggested functions of anemonefish bars include for camouflage, and aposematism for sea anemones (Merilaita and Kelley 2018). These are all non-mutually exclusive ideas and are worth further exploring, as animal color patterns can serve multiple roles depending on the receiver’s visual system and the environmental context they are viewed in (Marshall 2000b; Merilaita and Ruxton 2007; Gluckman and Cardoso 2010). This concept has relevance to UV cues which are usually one element of a complex pattern that is salient to UV-sensitive recipients, while supposedly inconspicuous to UV-blind predators, for example, reef sharks, grouper, barracudas (Siebeck and Marshall 2001; Siebeck et al. 2004). The application of a quantitative image analysis framework (e.g., QCPA; van den Berg et al. 2020) would provide a powerful tool to empirically test hypotheses regarding the function of anemonefish white bars.

## CONCLUSION

Our results show a novel function of UV signaling to confer social status in anemonefishes. The UV reflectance of *A. akindynos* skin strongly predicted the outcome of staged contests over dominance, and the level of aggression directed at lower ranked individuals. According to large differences in the UV reflectance and chromatic contrast of anemonefish skin, this could conceivably have a functional basis in juveniles for signaling submissiveness in family groups. This finding contributes to our understanding on the role that UV/color patterns can play in social contests and communication. It also promotes other lines of investigation including the importance of UV signaling in anemonefish long-term recruitment success, and the impact(s) that environmental degradation (e.g., sea anemone bleaching, turbidity) might have on the appearance and recognition of their color patterns.

We thank the Lizard Island Research Station directors and staff for their support. We thank Valerio Tettamanti, Abigail Shaughnessy, and Charlotte Lewis for their assistance in the field. We thank Kirsten Golding, Rowan Carew, Kyle Michie, and Abigail Shaughnessy for volunteering in the blind analysis of footage. We also thank both anonymous reviewers for their constructive comments that improved the final manuscript. Finally, we would like to acknowledge the Dingaal people as the traditional owners and custodians of Dyiigurra (Lizard Island).

## Supplementary Material

arac089_suppl_Supplementary_Material

## Data Availability

Analysis reported in this article can be reproduced using the data provided by Mitchell et al. (2022).
