# Supplementary material for: Higher ultraviolet skin reflectance signals submissiveness in the anemonefish, Amphiprion akindynos
Source: Behav Ecol. 2022 Nov 1;34(1):19–32. doi: 10.1093/beheco/arac089 (PMC9918861; doi:10.1093/beheco/arac089)
Supplement: arac089_suppl_Supplementary_Material [file arac089_suppl_supplementary_material.docx]

**
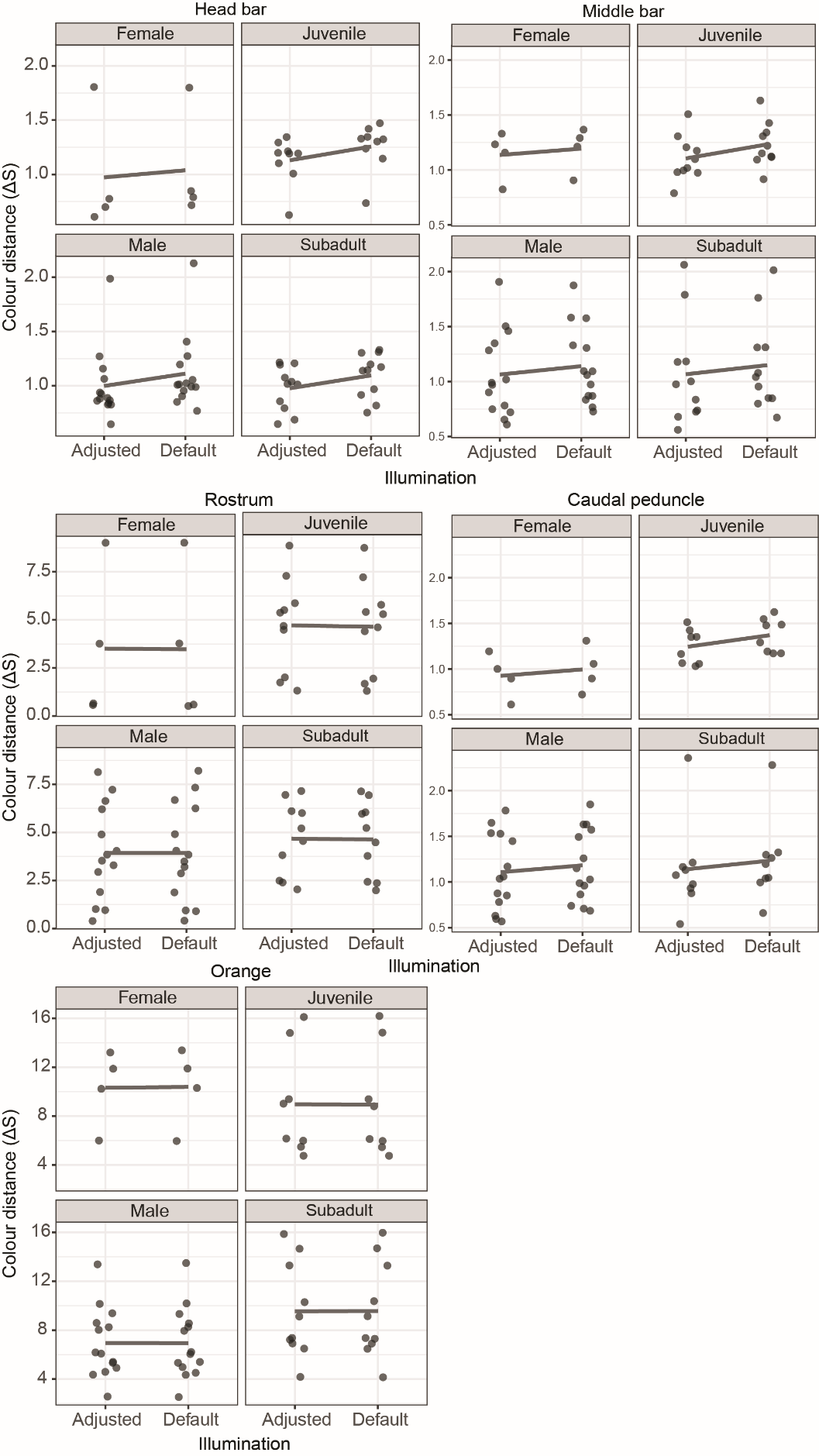
Supplementary Material**

**Supplementary Figure 1.** Colour distances (Δ*S*) between all skin patches and the white background of the aquarium for different life-stages when viewed in the ‘default’ UV-filter treatment and when ‘adjusted’ for the slight >450 nm absorbance induced by the slight yellowing of the filter. Lines denote shift in mean Δ*S*. Sample sizes give the number of individual measurements (points).


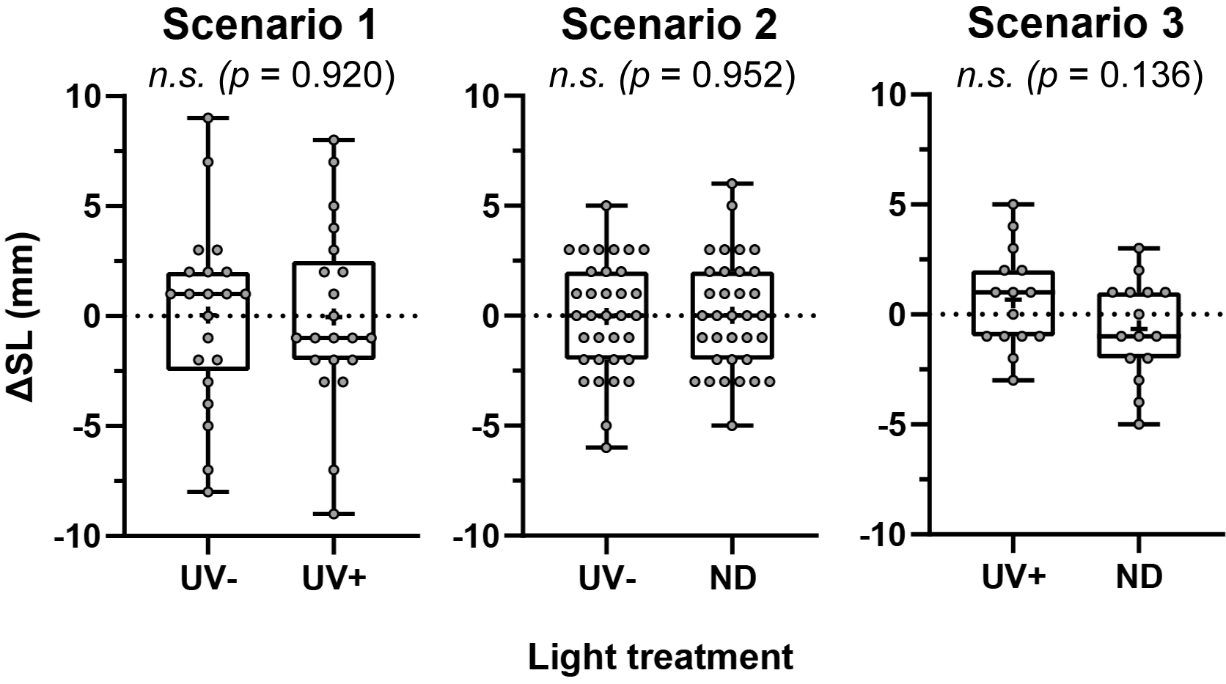


**Supplementary Figure 2.** Difference in standard length (ΔSL in mm) between anemonefish pairings in all three experimental scenarios. No significant (n.s.) differences (Wilcoxon signed rank test, *p* > 0.05) in ΔSL was found within any of the treatment combinations. Box plots show the median, 25^th^ and 75^th^ percentiles, and range indicated by the whiskers.

**Supplementary Table 1.** Outcomes of initial and repeated trials. Note that, repeat trials were not used for any downstream analyses as presented in the main text.

| Date | Fish ID 1 | Fish ID 1 state | Fish ID 2 | Fish ID 2 state | Treatment 1 | Treatment 2 | Winner |
| --- | --- | --- | --- | --- | --- | --- | --- |
| 31/06/2019 | 6 | Female | 2 | Male | ND-filter | No-filter | 6 |
| 3/07/2019 | 2 | Male | 6 | Female | ND-filter | No-filter | 2 |
|  |  |  |  |  |  |  |  |
| 29/06/2019 | 9 | Male | 7 | Female | UV-filter | No-filter | 9 |
| 1/07/2019 | 7 | Female | 9 | Male | UV-filter | No-filter | 7 |
|  |  |  |  |  |  |  |  |
| 28/06/2019 | 5 | Subadult | 3 | Male | UV-filter | No-filter | 5 |
| 1/07/2019 | 3 | Male | 5 | Subadult | UV-filter | No-filter | 3 |
|  |  |  |  |  |  |  |  |
| 1/07/2019 | 8 | Male | 13 | Male | UV-filter | No-filter | 13 |
| 2/07/2019 | 13 | Male | 8 | Male | UV-filter | No-filter | 8 |
|  |  |  |  |  |  |  |  |
| 30/06/2019 | 2 | Male | 7 | Female | UV-filter | No-filter | 2 |
| 2/07/2019 | 7 | Female | 2 | Male | UV-filter | No-filter | 7 |
|  |  |  |  |  |  |  |  |
| 2/07/2019 | 12 | Subadult | 18 | Subadult | UV-filter | No-filter | 12 |
| 3/07/2019 | 18 | Subadult | 12 | Subadult | UV-filter | No-filter | 18 |

**Supplementary Table 2.** Global GLMM for UV-filter – no-filter contests.

| Model terms | Estimate | Standard error | z value | Pr(>\|z\|) |
| --- | --- | --- | --- | --- |
| Intercept | 1.2242 | 0.7145 | 1.7130 | 0.0866 |
| Treatment | -2.4877 | 1.2552 | -1.982 | 0.0475* |
| Prior_wins | -0.1673 | 0.4061 | -0.4120 | 0.6803 |
| Prior_losses | -0.2339 | 0.4645 | -0.5030 | 0.6146 |
| Delta_mm | 0.2192 | 0.4119 | 0.5320 | 0.5946 |
| Aggression | 0.0839 | 0.4145 | 0.2020 | 0.8397 |

Treatment = light treatment, Prior_wins = no. of wins prior to the trial, Prior_losses = no. of losses prior to the trial, Delta_mm = difference in fish standard length, Aggression = sum of agonistic behaviour instances.

**Supplementary Table 3.** Top retained models selected with ΔAICc < 4 for no-filter – ND-filter contests.

| Model | Intercept | Treatment | Prior_wins | Prior_losses | Delta_mm | Aggression | Log-lik | df | AICc | Delta | Weight |
| --- | --- | --- | --- | --- | --- | --- | --- | --- | --- | --- | --- |
| 17 | 1.168 | + | NA | Na | NA | NA | -23.05 | 3 | 52.74 | 0.000 | 0.335 |
| 21 | 1.210 | + | NA | -0.2425 | NA | NA | -22.87 | 4 | 54.83 | 2.089 | 0.118 |
| 19 | 1.211 | + | NA | NA | -0.2425 | NA | -22.87 | 4 | 54.83 | 2.094 | 0.118 |
| 25 | 1.168 | + | -0.1972 | NA | NA | NA | -22.91 | 4 | 54.90 | 2.165 | 0.114 |
| 18 | 1.139 | + | 0.1285 | NA | NA | NA | -23.00 | 4 | 55.08 | 2.342 | 0.104 |

Note: only model 17 (the best model) was retained due to remaining models being nested models of the best and showing no improvement in the log-likelihood.

**Supplementary Table 4.** *Global GLMM for ND-filter – UV-filter contests.*

| Model terms | Estimate | Standard error | z value | Pr(>\|z\|) |
| --- | --- | --- | --- | --- |
| Intercept | -0.911 | 0.4225 | -2.155 | 0.0311* |
| Treatment | 1.815 | 0.642 | 2.827 | 0.005* |
| Prior_wins | -0.054 | 0.346 | -0.157 | 0.876 |
| Prior_losses | -0.065 | 0.364 | -0.178 | 0.859 |
| Delta_mm | -0.510 | 0.354 | -1.438 | 0.150 |
| Aggression | 0.060 | 0.284 | 0.212 | 0.832 |

Treatment = light treatment, Prior_wins = no. of wins prior to the trial, Prior_losses = no. of losses prior to the trial, Delta_mm = difference in fish standard length, Aggression = sum of agonistic behaviour instances.

**Supplementary Table 5.** *Top retained models selected with ΔAICc < 4 for ND-filter – UV-filter contests.*

| Model | Intercept | Treatment | Prior_wins | Prior_losses | Delta_mm | Aggression | Log-lik | df | AICc | Delta | Weight |
| --- | --- | --- | --- | --- | --- | --- | --- | --- | --- | --- | --- |
| 19  17  27  23  20  21 | -0.924  -0.876  -0.936  -0.908  -0.921  -0.841 | +  +  +  +  +  + | NA  NA  -0.088  NA  NA  NA | NA  NA  NA  -0.079  NA  -0.164 | -0.511  NA  -0.541  -0.497  -0.507  NA | NA  NA  NA  NA  0.071  NA | -39.57  -41.19  -39.53  -39.54  -39.54  -41.03 | 4  3  5  5  5  4 | 87.78  88.76  90.04  90.05  90.05  90.69 | 0.000  0.982  2.254  2.266  2.268  2.905 | 0.283  0.173  0.092  0.091  0.091  0.066 |
| 18 | -0.870 | + | NA | NA | NA | 0.123 | -41.10 | 4 | 90.83 | 3.051 | 0.062 |
| 25 | -0.875 | + | 0.045 | NA | NA | NA | -41.18 | 4 | 91.00 | 3.214 | 0.057 |

Note: models 27, 23, and 20 were excluded from the final averaged model due to being nested models of the best and showed no improvement in log-likelihood.

**Supplementary Table 6.** *Global GLMM for ND-filter – no-filter contests.*

| Model terms | Estimate | Standard error | z value | Pr(>\|z\|) |
| --- | --- | --- | --- | --- |
| Intercept | 0.237 | 0.782 | 0.303 | 0.762 |
| Treatment | -0.402 | 1.107 | -0.363 | 0.716 |
| Prior_wins | 0.057 | 0.695 | 0.083 | 0.934 |
| Prior_losses | -0.501 | 0.838 | -0.598 | 0.550 |
| Delta_mm | 0.360 | 0.545 | 0.660 | 0.509 |
| Aggression | -0.046 | 0.532 | -0.086 | 0.931 |

‘Treatment’ = light treatment, ‘Prior_wins’ = no. of wins prior to the trial, ‘Prior_losses’ = no. of losses prior to the trial, ‘Delta_mm’ = difference in fish standard length, ‘Aggression’ = sum of agonistic behaviour instances.

**Supplementary Table 7.** *Top retained models selected with ΔAICc < 4 for ND-filter – no-filter contests.*

| Model | Intercept | Treatment | Prior_wins | Prior_losses | Delta_mm | Aggression | Log-lik | df | AICc | Delta | Weight |
| --- | --- | --- | --- | --- | --- | --- | --- | --- | --- | --- | --- |
| 1  5 | 0.022  0.070 | NA  NA | NA  NA | NA  -0.561 | NA  NA | NA  NA | -20.50  -20.08 | 2  3 | 45.45  47.07 | 0.000  1.627 | 0.282  0.125 |
| 3 | 0.018 | NA | NA | NA | 0.249 | NA | -20.34 | 3 | 47.60 | 2.152 | 0.096 |
| 17  9  2  7 | 0.170  0.016  0.025  0.051 | +  NA  NA  NA | NA  -0.117  NA  NA | NA  NA  NA  -0.524 | NA  NA  NA  0.287 | NA  NA  0.056  NA | -20.44  -20.47  -20.49  -19.92 | 3  3  3  4 | 47.80  47.86  47.91  49.44 | 2.350  2.410  2.463  3.996 | 0.087  0.085  0.082  0.038 |

Note: models 9 and 2 were excluded from the final averaged model due to being nested models of the best and showed no improvement in log-likelihood.

**Supplementary Table 8.** *Global GLMM for agonistic behaviour assays*

| Model terms | Estimate | Standard error | z value | Pr(>\|z\|) |
| --- | --- | --- | --- | --- |
| Intercept | 2.72 | 0.40 | 6.88 | 6.17e-12 |
| Smallfishtreat | 0.69 | 0.30 | 2.34 | 0.019 |
| Deltasize | -0.16 | 0.10 | -1.56 | 0.12 |
| Fish1state | 0.19 | 0.34 | 0.56 | 0.58 |
| Fish2state | -0.11 | 0.37 | -0.30 | 0.77 |

‘Smallfishtreat’ = light treatment of small fish, ‘Deltasize’ = standard length difference, ‘Fish1state’ = life-stage of fish in UV-filter treatment, ‘Fish2state’ = life-stage of fish in ND-filter treatment.

**Supplementary Table 9.** *Top retained models selected with ΔAICc < 4 for agonistic behaviour assays.*

| Model | Intercept | Treatment | Delta_size | Fish1state | Fish2state | Log-lik | df | AICc | Delta | Weight |
| --- | --- | --- | --- | --- | --- | --- | --- | --- | --- | --- |
| 9 | 2.59 | + | NA | NA | NA | -147.2 | 5 | 306.1 | 0.00 | 0.357 |
| 10 | 2.76 | + | -0.107 | NA | NA | -146.3 | 6 | 307.1 | 0.97 | 0.220 |
| 13 | 2.58 | + | NA | NA | + | -147.2 | 6 | 308.9 | 2.74 | 0.091 |
| 11 | 2.56 | + | NA | + | NA | -147.2 | 6 | 308.9 | 2.76 | 0.090 |
| 12 | 2.63 | + | -0.145 | + | NA | -145.9 | 7 | 309.2 | 3.07 | 0.077 |
| 2 | 3.13 | NA | -0.269 | NA | NA | -148.9 | 5 | 309.5 | 3.41 | 0.065 |

Note: models 13 and 11 were excluded from the final averaged model due to being nested models of the best and showed no improvement in log-likelihood.

**Supplementary Table 10.** Relative quantum catches (Q_i_) for anemonefish skin viewed under the three experimental light treatments.

| **SUMMARY OF RELATIVE QUANTUM CATCHES** | | | | |
| --- | --- | --- | --- | --- |
| **No filter** |  | **Average QCs** | | |
| Stage | Skin | Q1 | Q2 | Q3 |
| Juvenile | Head stripe | 0.38 | 0.34 | 0.28 |
| Juvenile | Middle stripe | 0.38 | 0.34 | 0.28 |
| Juvenile | Caudal | 0.38 | 0.33 | 0.27 |
| Juvenile | Orange | 0.20 | 0.33 | 0.47 |
| Juvenile | Rostrum | 0.26 | 0.36 | 0.38 |
| Subadult | Head stripe | 0.36 | 0.34 | 0.30 |
| Subadult | Middle stripe | 0.34 | 0.35 | 0.31 |
| Subadult | Caudal | 0.36 | 0.34 | 0.30 |
| Subadult | Orange | 0.18 | 0.32 | 0.51 |
| Subadult | Rostrum | 0.26 | 0.33 | 0.41 |
| Male | Head stripe | 0.35 | 0.35 | 0.30 |
| Male | Middle stripe | 0.33 | 0.36 | 0.31 |
| Male | Caudal | 0.35 | 0.35 | 0.31 |
| Male | Orange | 0.20 | 0.32 | 0.48 |
| Male | Rostrum | 0.30 | 0.32 | 0.38 |
| Female | Head stripe | 0.33 | 0.35 | 0.32 |
| Female | Middle stripe | 0.32 | 0.36 | 0.32 |
| Female | Caudal | 0.33 | 0.35 | 0.31 |
| Female | Orange | 0.16 | 0.30 | 0.54 |
| Female | Rostrum | 0.33 | 0.31 | 0.36 |
| **UV filter** |  | **Average QCs** | | |
| Stage | Skin | Q1 | Q2 | Q3 |
| Juvenile | Head stripe | 0.35 | 0.35 | 0.30 |
| Juvenile | Middle stripe | 0.35 | 0.35 | 0.30 |
| Juvenile | Caudal | 0.36 | 0.35 | 0.29 |
| Juvenile | Orange | 0.14 | 0.35 | 0.51 |
| Juvenile | Rostrum | 0.23 | 0.37 | 0.40 |
| Subadult | Head stripe | 0.34 | 0.36 | 0.30 |
| Subadult | Middle stripe | 0.33 | 0.36 | 0.31 |
| Subadult | Caudal | 0.34 | 0.35 | 0.31 |
| Subadult | Orange | 0.13 | 0.33 | 0.54 |
| Subadult | Rostrum | 0.22 | 0.35 | 0.43 |
| Male | Head stripe | 0.33 | 0.36 | 0.31 |
| Male | Middle stripe | 0.32 | 0.36 | 0.32 |
| Male | Caudal | 0.33 | 0.36 | 0.31 |
| Male | Orange | 0.16 | 0.33 | 0.50 |
| Male | Rostrum | 0.26 | 0.34 | 0.41 |
| Female | Head stripe | 0.32 | 0.36 | 0.32 |
| Female | Middle stripe | 0.31 | 0.37 | 0.32 |
| Female | Caudal | 0.32 | 0.36 | 0.32 |
| Female | Orange | 0.11 | 0.32 | 0.57 |
| Female | Rostrum | 0.29 | 0.33 | 0.38 |
| **ND filter** |  | **Average QCs** | | |
| Stage | Skin | Q1 | Q2 | Q3 |
| Juvenile | Head stripe | 0.38 | 0.34 | 0.28 |
| Juvenile | Middle stripe | 0.38 | 0.34 | 0.28 |
| Juvenile | Caudal | 0.38 | 0.33 | 0.29 |
| Juvenile | Orange | 0.20 | 0.33 | 0.47 |
| Juvenile | Rostrum | 0.26 | 0.36 | 0.38 |
| Subadult | Head stripe | 0.36 | 0.34 | 0.30 |
| Subadult | Middle stripe | 0.34 | 0.35 | 0.31 |
| Subadult | Caudal | 0.36 | 0.34 | 0.30 |
| Subadult | Orange | 0.18 | 0.32 | 0.51 |
| Subadult | Rostrum | 0.26 | 0.33 | 0.41 |
| Male | Head stripe | 0.35 | 0.35 | 0.30 |
| Male | Middle stripe | 0.33 | 0.36 | 0.31 |
| Male | Caudal | 0.35 | 0.35 | 0.31 |
| Male | Orange | 0.20 | 0.32 | 0.48 |
| Male | Rostrum | 0.30 | 0.31 | 0.38 |
| Female | Head stripe | 0.33 | 0.35 | 0.32 |
| Female | Middle stripe | 0.32 | 0.36 | 0.32 |
| Female | Caudal | 0.33 | 0.35 | 0.32 |
| Female | Orange | 0.16 | 0.30 | 0.54 |
| Female | Rostrum | 0.33 | 0.31 | 0.36 |

‘Q1’ = SWS, ‘Q2’ = MWS, and ‘Q3’ = LWS.

**Camera setup used for underwater photography**

Underwater photographs of anemonefish (*Amphiprion akindynos*) were taken on patch reefs near Lizard Island on the Great Barrier Reef. Photographs in the visible (RGB) were captured using an Olympus (TG-5) camera (with Nikon 60 mm Micro), while a UV-converted Nikon (D810) fitted with both a short-pass filter (UG 11) and far-red filter took UV images.

**Anemone and reef light measurements**

Sea anemone (*Stichodactyla gigantea*) reflectance was based on averaged (n = 10) measurements recorded in-situ using a submersible spectrometer (USB2000 Ocean Optics) with a 100µm fibre and relative to a 99% Spectralon reflectance standard positioned next to the anemone. Reflectance measurements of tentacles used natural daylight as a light source at midday during non-overcast conditions at ~3m depth.

Downwelling spectral irradiance measurements were taken on the reef (n = 3) using a 100µm fibre with a cosine corrector and a submersible spectrometer (USB2000 Ocean Optics) at ~5m depth.
